# Efficient Glycolysis of Polyethylene Terephthalate (PET) Catalyzed by Cyclic(alkyl)(amino)carbene Copper Complexes

**DOI:** 10.3390/molecules30234521

**Published:** 2025-11-23

**Authors:** Lei Zhou, Irfan Purnawan, Nurul Hidayati Fithriyah, Mingxin Li, Hao Huang, Jiaqin He, Yuanyou Wang

**Affiliations:** 1 School of Chemical Engineering, Yangzhou Polytechnic Institute, Yangzhou 225127, China; 18362929192@163.com (M.L.); huangh0825@163.com (H.H.); hejq@ypi.edu.cn (J.H.); 2 Jiangsu Polyester Synthesis and Renewable Technology Engineering Research Center, Yangzhou 225127, China; 3 Chemical Engineering Department, Universitas Muhammadiyah Jakarta, Jakarta 10510, Indonesia; irfan.purnawan@umj.ac.id (I.P.); nurul.fithriyah@umj.ac.id (N.H.F.)

**Keywords:** PET, glycolysis, cyclic (alkyl)(amino)carbenes, recycling

## Abstract

Polyethylene terephthalate (PET) is widely used, yet the accumulation of its waste poses serious environmental challenges, making efficient recycling essential. PET glycolysis using EG as a solvent has emerged as a green recycling strategy. In this study, a cyclic alkylamino carbene copper (CAAC-Cu) complex was prepared as a catalyst for PET glycolysis. Under optimized conditions (160 °C, 90 min, catalyst amount 3 wt%, and PET/EG = 1:4.), PET conversion reached 98.2%, the selectivity toward BHET was 88.1%, and the yield was 86.5%. Kinetic analysis indicated that the glycolysis follows first-order kinetics with an activation energy of 98.7 kJ mol^−1^. In addition, the catalyst can be recovered together with excess EG, and after multiple recycles, PET degradation remained above 95% and BHET yield remained above 80%. A possible mechanism has also been proposed: Cu acts as a Lewis acid coordinating to the carbonyl oxygen of PET, facilitating ester bond activation, while the amino-carbene forms hydrogen bonds with EG, assisting bond cleavage in a Brønsted-base manner. This catalytic system provides a novel and efficient approach for the green, high-performance glycolysis of PET.

## 1. Introduction

Polyethylene terephthalate (PET), a high-performance polyester valued for its excellent mechanical properties, chemical stability, and processability, has extensive global applications. Consequently, the accumulation of PET waste poses a significant environmental challenge. Within the global context of “carbon neutrality” and the “circular economy,” efficient recycling and resource utilization of post-consumer PET have garnered widespread attention. While physical recycling via melt-processing and pelletization allows for PET reuse, the resulting material often suffers from degraded properties due to chain scission and impurity introduction. In contrast, chemical recycling, which operates at the molecular level, offers a pathway to higher-value products [1,2,3].

Chemical degradation of PET primarily proceeds through alcoholysis, hydrolysis, or ammonolysis [4,5,6]. PET alcoholysis typically employs ethylene glycol, methanol and iso-octanol as solvents, generating bis(2-hydroxyethyl) terephthalate (BHET), dimethyl terephthalate (DMT), or dioctyl terephthalate (DOTP), respectively. Among these, glycolysis, which depolymerizes the polyester into high-purity monomers via transesterification, is particularly prominent. BHET is the precursor for PET, sharing an identical molecular structure. Compared to the limitations of DMT, which requires multi-step purification including hydrolysis, and DOTP, which is hindered for direct polycondensation due to terminal group sterics, the ethylene glycol route has emerged as a research focus for green recycling technologies due to its superior atom economy and product re-polymerizability [7,8,9].

Recent research on PET glycolysis catalysts has concentrated on performance and stability. Non-metallic catalysts, such as functionalized ionic liquids and deep eutectic solvents, are notable for their environmental friendliness [10,11]. The mechanism primarily relies on hydrogen-bond activation or synergistic acid–base effects to cleave the ester bond. However, these systems often exhibit low intrinsic activity, necessitating prolonged reaction times and frequently requiring intensification by external fields like microwave or ultraviolet irradiation [12,13]. In comparison, transition metal catalysts demonstrate higher efficiency by leveraging the d-orbital electrons of the metal center to coordinate strongly with the PET ester group, polarizing the carbonyl electron cloud and thereby activating the ester moiety. Liu et al. reported that when zinc acetate/1,3-dimethylurea (Zn(OAc)_2_/DMU) is used for the PET glycolysis, Zn^2+^ coordinates with the ester bonds, and the DMU cation stabilizes the intermediates through electrostatic interaction, thus forming a synergistic reaction pathway [14]. Similarly, in the Urea/ZnCl_2_ system, Zn^2+^ coordination combines with the hydrogen-bonding network from urea to lower the reaction activation energy [13]. Our prior studies using Zn(OAc)_2_/ChCl for PET degradation confirmed that the metal center coordinates with the ester group, facilitating a five-membered ring transition state that accelerates ester bond cleavage [15,16].

The performance of transition metal catalysts is highly dependent on ligand design and selection. Cyclic (alkyl)(amino)carbenes (CAACs), highly reactive intermediates with two non-bonding electrons, exhibit strong nucleophilicity (higher HOMO, stronger σ-donor) and electrophilicity (lower LUMO, stronger π-acceptor) [17,18]. Their exceptional ability to stabilize highly reactive species and metal centers, enabling the activation of strong bonds and small molecules, ranks them among the most powerful organic ligands in catalysis [19,20]. Our previous work successfully applied CAAC-metal complexes in diverse catalytic reactions, revealing synergistic electronic effects between the CAAC ligand and the metal center that effectively promote C=O bond activation or hydrogen transfer processes [21]. In this study, the CAAC-Cu catalyst was successfully prepared starting from 2,6-diisopropylaniline, with copper(I) chloride serving as the metal center. Various reaction parameters including catalyst loading and temperature were optimized, and a plausible reaction mechanism was proposed. The experimental results demonstrated that the CAAC-Cu catalyst exhibits excellent activity for PET glycolysis, and indicated that the reaction follows first-order reaction kinetics. In addition, the catalysts can be conveniently recycled alongside the reaction solvent for repeated use.

## 2. Results

### 2.1. Synthesis of CAAC-Cu Complex

**Scheme 1:**
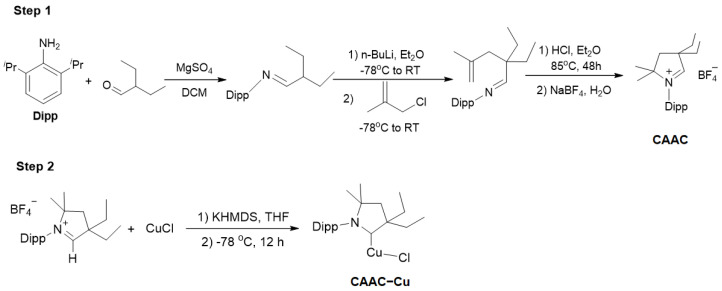
Synthesis of CAAC-Cu complex.

Firstly, the CAAC ligand was prepared following Scheme 1, and the ligand was purified using diethyl ether, which was obtained as a white solid after vacuum drying. Subsequently, the CAAC-Cu complex was synthesized as a white solid following previously reported protocols [22]. The NMR results were consistent with the reported data, which were used to deduce the catalyst structure. The results indicated that in the ^1^H NMR spectrum of the CAAC-Cu complex, the original signal at δ 9.7 ppm of the ligand disappeared, while a distinct carbene carbon appeared at δ 249.5 ppm in the ^13^C NMR spectrum, confirming the successful preparation of the catalyst. More detailed information can be found in the ESI (Supplementary Materials).

**Figure 1:**
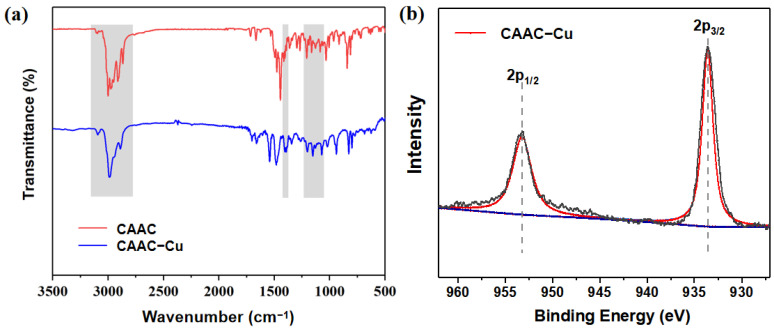
(**a**) FT-IR spectrum for the CAAC and CAAC−Cu complex; (**b**) XPS spectrum of Cu(I) in the CAAC−Cu complex.

Figure 1a displays the FT-IR results for the CAAC-Cu catalyst; the peaks at 3095 cm^−1^, 2980 cm^−1^, and 2887 cm^−1^ are attributed to C-H stretching vibrations of -CH, -CH_2_, or -CH_3_. The peaks at 1540 cm^−1^ and 1485 cm^−1^ correspond to aromatic ring C-C skeletal vibrations, and the 1344 cm^−1^ peak to aromatic ring C-N vibration. Due to the changes in the electronic environment around the N-C bonds before and after metal coordination, the frequency is mainly affected by the electronic and steric effects of adjacent groups. So, the heterocyclic N-C vibration peak exhibited a slight shift from 1412 cm^−1^ to 1400 cm^−1^. Peaks at 1202 cm^−1^, 1152 cm^−1^, and 1070 cm^−1^ are ascribed to side-chain C-C skeletal vibrations. In addition, XPS surface analysis of Cu(I) for the CAAC-Cu complex is presented in Figure 1b. The binding energy (BE) of surface charges was calibrated using the C 1s peak of carbon at 284.5 eV. It can be seen that the sample exhibits a Cu 2p_3_/_2_ peak at 932.9 eV and a Cu 2p_1_/_2_ peak at 952.8 eV, both of which are characteristic of Cu(I) compounds, as verified by comparison with standard spectral data [23,24]. Notably, no characteristic satellite peaks of Cu(II) (~942 eV or ~962 eV) were detected, which confirms the stability of the Cu(I) valence state in the complex. Collectively, these results further demonstrate the structural integrity of the carbene moiety during synthesis and the successful coordination of the metal active center.

### 2.2. General Procedure for the PET Glycolysis

In each PET degradation experiment, EG was used as the reaction medium with the CAAC-Cu complex as the catalyst. All degradation reactions were carried out in a 250 mL double-necked round-bottom glass reactor equipped with a magnetic stirrer, thermometer, and reflux condenser to ensure stable and controllable reaction conditions.

The experiments were performed under atmospheric pressure, with key parameters such as reaction time, temperature, and catalyst dosage systematically optimized to explore the optimal reaction conditions. Upon completion of the reaction, the system was cooled to 100 °C and subjected to hot filtration to separate unreacted PET. After the filtrate was completely cooled, a secondary filtration was performed, and the resulting filter residue was BHET. At this point, the CAAC-Cu complex remained in the excess EG and could be directly reused for cyclic reactions. The filter residue was dissolved in an appropriate amount of hot water, and after cooling and crystallization, high-purity BHET products were obtained as white needle-like crystals.

The separation yield of BHET was calculated, and its structure was determined by ^1^H NMR spectroscopy (Figure S5), DSC analysis (Figure S8), and elemental analysis (Table S1), confirming the main product is BHET. The conversion rate of PET, selectivity, and yield of BHET were calculated according to the following Equations (1)–(3):

(1) $$Conversion\;of\;PET=\frac{W_{0}-W_{1}}{W_{0}}\times 100\%$$

(2) $$Selectivity\;of\;BHET=\frac{n_{BHET}}{n_{units}}\times 100\%$$

(3) $$Yield\;of\;BHET=S_{BHET}\times C_{PET}$$

where W_0_ represents the initial weight of PET and W_1_ represents the weight of undegraded PET. n_BHET_ represents the number of moles of BHET, n_units_ represents the number of moles of the degraded PET units, S_BHET_ represents the selectivity of BHET, and C_PET_ represents the conversion of BHET. The molecular weight of PET is calculated based on its repeat unit and is approximately 192 g/mol [25].

### 2.3. CAAC-Cu Performance in PET Glycolysis

**Figure 2:**
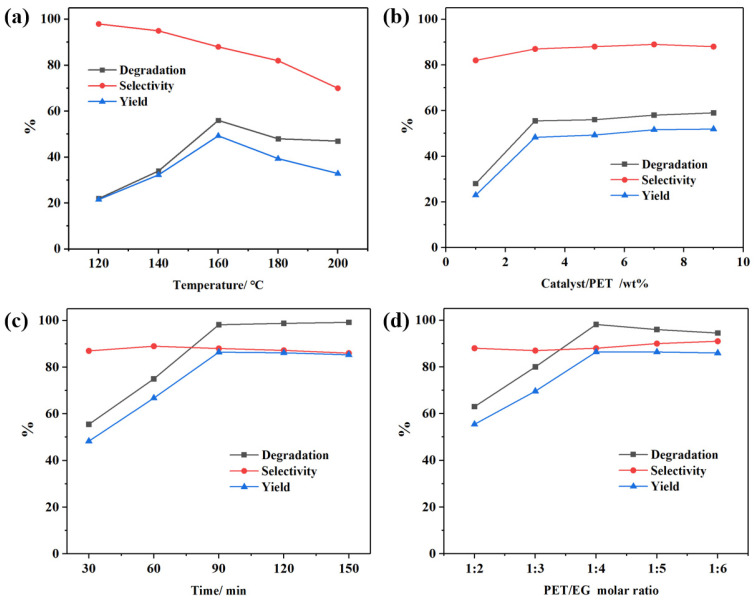
(**a**) Effect of temperature. Reaction conditions: 30 min, 5 wt% catalyst, PET/EG = 1:4 molar ratio. (**b**) Effect of catalyst dosage. Reaction conditions: 160 °C, 30 min, PET/EG = 1:4 molar ratio. (**c**) Effect of time, reaction conditions: 160 °C, 3 wt% catalyst, PET/EG = 1:4 molar ratio. (**d**) Effect of EG amount, reaction condition: 160 °C, 90 min, 3 wt% catalyst.

The influence of reaction temperature on PET glycolysis was investigated under specific conditions (5 g PET, 30 min, PET/EG molar ratio of 1:4) using a 5 wt% CAAC-Cu complex as catalyst, with the results presented in Figure 2a. At 120 °C, the PET glycolysis rate was minimal, achieving a BHET yield of 22.3%, while the selectivity toward BHET was relatively higher at this low temperature; as the temperature increased from 120 °C to 160 °C, both the PET glycolysis rate and Y_BHET_ increased, though this was accompanied by a concurrent decrease in S_BHET_. Optimal PET C_PET_ and Y_BHET_ were obtained at 160 °C, reaching 56.2% and 49.3%, respectively. Further temperature elevation did not significantly improve C_PET_, because the CAAC-Cu complex undergoes slight decomposition when the temperature exceeds 150 °C from the TGA (Figure S7), which leads to a decrease in its catalytic activity. In addition, excessively high temperature enhanced side reactions instead, leading to reduced Y_BHET_ and S_BHET_. Hence, the subsequent experiments were conducted at 160 °C.

Then, to explore the impact of catalyst dosage on PET glycolysis, experiments were carried out under these conditions: 5 g PET, 30 min, 160 °C, and a PET/EG molar ratio of 1:4, with the results depicted in Figure 2b. It was observed that at a catalyst loading of 3 wt%, the degradation rate approached its maximum with C_PET_ and Y_BHET_ reaching 55.8% and 48.7%, respectively. Further increasing the catalyst amount did not alter the degradation rate. This confirms that the CAAC-Cu complex still exhibits good catalytic activity even at relatively low concentrations.

The influence of reaction time on PET glycolysis was examined under the conditions of 5 g PET, 3 wt% catalyst, a PET/EG molar ratio of 1:4, and 160 °C, with the results presented in Figure 2c. With extension of the reaction time, the conversion of PET increased significantly until it approached nearly 100%. The S_BHET_ and Y_BHET_ reached the maximum values of 87.8% and 86.4% when the reaction time was 90 min. Beyond 90 min, a slight decline in S_BHET_ and Y_BHET_ was observed, attributed to the equilibrium established between BHET, dimers, and oligomers in the PET glycolysis reaction [26].

Finally, the influence of the reactant ratio was investigated under the previously optimized reaction conditions (5 g PET, 3 wt% catalyst, 90 min reaction time, 160 °C), with the results presented in Figure 2d. The experimental data showed that when the dosage of EG was relatively low, the reaction efficiency was low, which is attributed to the fact that PET degradation is a heterogeneous reaction. So, increasing the concentration of substrates can effectively accelerate the reaction rate. When the molar ratio of EG to PET reached 4:1, all performance indicators achieved optimal values. However, a further increase in EG dosage led to a slight decline in reaction performance, possibly due to the reduced concentration of reactants and the increased occurrence of side reactions. Therefore, the optimal PET/EG molar ratio was determined to be 1:4.

**Figure 3:**
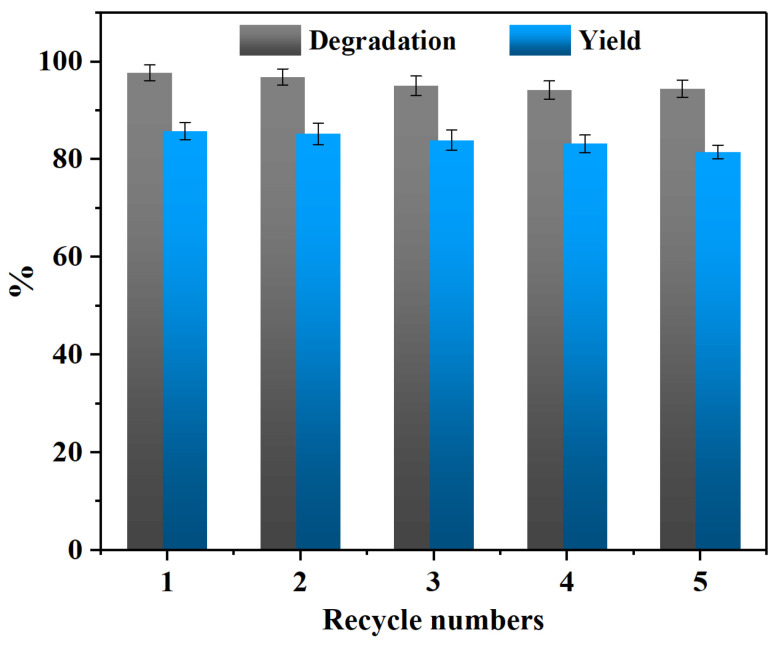
Recycling of CAAC-Cu in PET glycolysis (160 °C, 90 min, 3 wt% catalyst, PET/EG = 1:4 molar ratio).

By comparing the above results, it can be observed that the reaction conditions exert a significant influence on the PET glycolysis reaction. Considering the cost of practical application comprehensively, the optimal reaction conditions for PET glycolysis using the CAAC-Cu catalyst are as follows: 160 °C, 90 min, catalyst dosage of 3 wt%, and a PET/EG molar ratio of 1:4. The PET conversion, BHET selectivity, and yield reached 98.2%, 88.1%, and 86.5%, respectively. This study focuses solely on the CAAC-Cu catalyst because the existing research has shown that residual catalysts inherently present in polymers also exert a significant impact on the degradation process. Nevertheless, the PET used in this experiment is from the same batch, so this influence could be temporarily eliminated [27]. In addition, the reusability of CAAC-Cu was also investigated. After each experiment, the catalyst was recovered together with the excess EG, and only the consumed EG needed to be supplemented, which is very convenient. As shown in Figure 3, after multiple recovery cycles, the catalytic activity remains good, with the PET degradation rate and BHET yield maintained above 90% and 80%, respectively. More detailed information can be found in Table S2.

### 2.4. Kinetic of PET Glycolysis Catalyzed by CAAC-Cu

In the field of polymer depolymerization kinetics, there is an ongoing discussion that the reaction order is typically regarded as first-order [28,29]. Accordingly, for the glycolysis of PET catalyzed by CAAC-Cu, the reaction was initially assumed to follow first-order kinetic equations:

(4) $$\;\;\;\frac{d(C_{PET})}{dt}=-kC_{PET}$$

Here, k denotes the reaction rate constant, while CPET represents the concentration of PET at time t.

(5) $$C_{PET}=C_{PET0}\left(1-x\right)$$

Here, x refers to the conversion of PET. Subsequently, Equation (4) can be rearranged to give:

(6) $$\frac{dx}{dt}=k\left(1-x\right)$$

Equation (6) was integrated over time to provide:

(7) $$ln\frac{1}{1-x}=kt$$

The activation energy (Ea) can be derived from the Arrhenius equation using the determined rate constant. Here, A denotes the pre-exponential factor, R is the gas constant with a value of 8.314 J K^−1^ mol^−1^, and T represents the temperature in Kelvin.

(8) $$\;\;lnk=-\frac{E_{a}}{RT}+lnA$$

**Figure 4:**
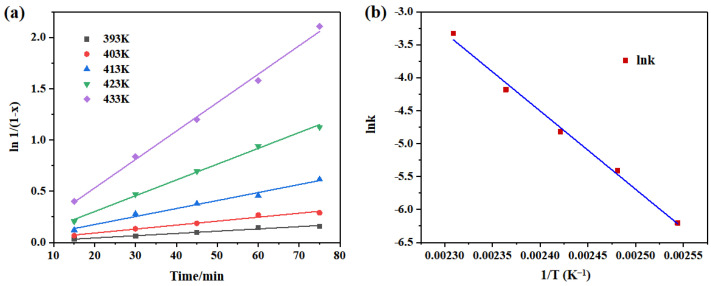
(**a**) Effect of temperature on the rate of PET glycolysis. Reaction conditions: 3 wt% catalyst, PET/EG = 1:4 molar ratio. (**b**) Arrhenius plots of the rate constant of PET glycolysis.

The effect of reaction temperature on the degradation rate of PET in the presence of CAAC-Cu is shown in Figure 4a. This indicates that the reaction conversion rate is proportional to the reaction time at different temperatures, and the process is a first-order kinetic reaction. In addition, EG can penetrate PET, disrupt intermolecular force, and induce swelling, accelerating depolymerization [30]. Elevated temperature enhances EG permeability and weakens PET chain interactions, boosting swelling significantly. The kinetics data show a mild rate increase at low temperatures; beyond a threshold, swelling fully acts to sharply accelerate degradation. As PET degradation is mass-transfer-controlled, swelling expands the reactant contact area and improves efficiency [31]. The activation energy of this reaction, calculated from the slope of the Arrhenius plot in Figure 4b, is 98.7 kJ·mol^−1^. Furthermore, with the increase in temperature, the reaction rate constant increases rapidly, which demonstrates that temperature exerts a significant influence on PET degradation, and this result is consistent with previous experiments.

### 2.5. Proposed Mechanism of PET Glycolysis

In the process of PET glycolysis or other transesterification reactions, transition metals are generally regarded as Lewis acid catalysts, with the capability to attack the carbonyl oxygen attached to the ester group [32,33]. In this study, a possible mechanism for the degradation of PET catalyzed by CAAC-Cu was proposed based on previous studies [10,14,34]. The amino group in CAAC-Cu can form a hydrogen bond with EG. This subsequently enhances the electronegativity of the hydroxyl oxygen in EG, facilitating its attack on the carbonyl group of PET. Meanwhile, Cu^1+^ acts as a Lewis acid, protonating the carbonyl group of the ester, strengthening the electrophilicity of the carbon atom in the carbonyl group. This further makes the carbonyl group of PET more susceptible to nucleophilic attack by EG, and the acid–base synergistic catalytic effect may contribute to PET glycolysis. However, although PET can be almost completely degraded, oligomers and dimers remain in the products due to the reaction having reached equilibrium. The molecular weights of these oligomers and dimers are generally between 500 and 900, which has been confirmed in previous studies [9].

## 3. Materials and Methods

### 3.1. Materials

PET pellets (2 × 2 × 2.5 mm, Mw 20–30 kDa) were purchased from Sinopec Yizheng Chemical Fiber Co., Ltd. (Yangzhou, China). After being ground in a crusher, these PET pellets were sieved through a screen to collect powders with a particle size of 60–80 mesh, which were then used for the degradation experiment. Ethylene glycol (EG), 2,6-Diisopropylaniline (Dipp), copper(I) chloride, n-Butyllithium, Tetrahydrofuran (THF), ethyl acetate, and potassium bis (trimethylsilyl) amide (KHMDS) were purchased from Sinopharm Chemical Reagent Co., Ltd. (Shanghai, China), Shanghai Macklin Biochemical Technology Co., Ltd. (Shanghai, China) and Shanghai Aladdin Biochemical Technology Co., Ltd. (Shanghai, China). All reagents were used as received without further treatment.

### 3.2. Characterization of Catalysts and Products

The prepared CAAC-Cu catalyst and the PET glycolysis products were characterized. ^1^H and ^13^C spectra were recorded on a Bruker Avance 400 spectrometer (Landkreis Karlsruhe, Baden-Württemberg, Germany). NMR multiplicities are abbreviated as follows: s = singlet, d = doublet, t = triplet, q = quartet, m = multiplet, br = broad signal. Fourier transform-infrared (FT-IR) spectra were obtained with a Nicolet 380 (Thermo Fisher Scientific, Waltham, MA, USA) spectrometer, using KBr as the blank. Differential scanning calorimetry (DSC) scans were obtained using the DSC-1 STARe system by heating from 25 °C to 180 °C at a heating rate of 10 °C min^−1^ in an atmosphere of nitrogen with a flow rate of 10 mL min^−1^ (Mettler Toledo, Zurich, Switzerland). Mass spectroscopy (MS) measurements were performed on a Thermo Finnigan Q-TOF spectrometer (San Jose, CA, USA). X-ray photoelectron spectroscopy (XPS) analysis was performed by PHI 5000 Versa Probe (ULVAC-PHI, Kanagawa, Japan). Inductively coupled plasma optical emission spectrometer (ICP-OES) AVIO 200 (PerkinElmer, Waltham, MA, USA). HPLC analysis of the main product was performed with a column temperature of 25 °C, a detector temperature of 40 °C, a solvent water/methanol ratio of 6:4, and a flow rate of 0.3 mL/min. We used an ACQUITY HPLC (Waters, Milford, MA, USA) which was equipped with a refractive index detector and BET C18 column. Thermogravimetric analysis (TGA) was performed using DTG-60H under a nitrogen atmosphere by heating the sample from 25 °C to 600 °C at a rate of 5 °C min^−1^ (Shimadzu, Kyoto, Japan).

## 4. Conclusions

In summary, a cyclic(alkyl)(amino)carbene copper (CAAC-Cu) complex catalyst was successfully prepared and applied for the first time in the glycolysis of PET, realizing efficient degradation and high-value conversion of PET. Multiple characterizations have confirmed the successful coordination between the CAAC ligand and CuCl as well as the stable existence of the metal active center. The optimal parameters were determined to be a reaction temperature of 160 °C, a reaction time of 90 min, a catalyst dosage of 3 wt%, and a PET/EG molar ratio of 1:4, under which the PET conversion rate reached 98.2%, the selectivity for BHET was 88.1%, and the yield was 86.5%. In addition, the experimental results showed the reaction followed first-order kinetics with an activation energy of 98.7 kJ·mol^−1^. The CAAC-Cu catalyst could be recovered with the filtrate and maintained high catalytic activity after multiple reaction cycles. A plausible reaction mechanism has been inferred as the copper ions attack the ester groups on PET to form carbocations (acid-catalyzed pathway), while the amino carbene formed hydrogen bonds with EG to assist ester bond cleavage (base-catalyzed pathway). In conclusion, the CAAC-Cu catalyst provides a novel strategy for PET chemical recycling and holds great significance for the industrialization of closed-loop PET recycling, mitigation of plastic pollution, and achievement of “carbon neutrality”.

## Data Availability

The original contributions presented in this study are included in the article/Supplementary Material. Further inquiries can be directed to the corresponding authors.

## Supplementary Materials

The following supporting information can be downloaded at: https://www.mdpi.com/article/10.3390/molecules30234521/s1, Figure S1. ^1^H NMR spectrum of CAAC; Figure S2. ^13^C NMR spectrum of CAAC; Figure S3. ^1^H NMR spectrum of CAAC-Cu; Figure S4. ^13^C NMR spectrum of CAAC-Cu; Figure S5. ^1^H NMR spectrum of the main product; Figure S6. XPS spectrum of CAAC-Cu was calibrated using the C 1s peak of carbon at 284.5 eV; Figure S7. TGA curves of CAAC-Cu; Figure S8. DSC curves of main product; Figure S9. Mass spectrum of main product; Table S1. Elemental analysis results of the main product; Table S2. Recycling experiment data.

## References

[1] Cao J., Liang H., Yang J., Zhu Z., Deng J., Li X., Elimelech M., Lu X. (2024). Depolymerization mechanisms and closed-loop assessment in polyester waste recycling. Nat. Commun..

[2] Dong Q., Lele A.D., Zhao X., Li S., Cheng S., Wang Y., Cui M., Guo M., Brozena A.H., Lin Y. (2023). Depolymerization of plastics by means of electrified spatiotemporal heating. Nature.

[3] Savage P.E. (2024). Renewable fuels and chemical recycling of plastics via hydrothermal liquefaction. Acc. Chem. Res..

[4] Abedsoltan H. (2023). A focused review on recycling and hydrolysis techniques of polyethylene terephthalate. Polym. Eng. Sci..

[5] Gan Y., Tang J.-T., Li X., Nie X.-S., Ye B. (2025). Valorization of polyester plastics and biomass into amines through a dual zirconium catalysis. ACS Sustain. Chem. Eng..

[6] Kopperi H., Mamidi V., Suresh G., Mohan S.V. (2025). Tandem chemical hydrolysis and bioelectrochemical upcycling of waste polyethylene terephthalate (PET) for sustainable biobutanol and ethanol production ensuring plastics circularity. Green. Chem..

[7] Luo X., Li Q., Chen X. (2025). Mechanistic investigation on hydrolysis, alcoholysis, and ammonolysis of polyethylene terephthalate initiated by participation of calcium ions. Process Saf. Environ..

[8] Li F., Yao X., Ding R., Bao Y., Zhou Q., Yan D., Li Y., Xu J., Xin J., Lu X. (2024). Directional glycolysis of waste PET using deep eutectic solvents for preparation of aromatic-based polyurethane elastomers. Green Chem..

[9] Zhou L., Qin E., Huang H., Wang Y., Li M. (2024). PET glycolysis to bhet efficiently catalyzed by stable and recyclable Pd-Cu/γ-Al_2_O_3_. Molecules.

[10] Liu Y., Yao X., Yao H., Zhou Q., Xin J., Lu X., Zhang S. (2020). Degradation of poly(ethylene terephthalate) catalyzed by metal-free choline-based ionic liquids. Green Chem..

[11] Ha G.-S., Al Mamunur Rashid M., Ha J.-M., Yoo C.-J., Jeon B.-H., Jeong K., Kim K.H. (2024). Enhancing polyethylene terephthalate conversion through efficient microwave-assisted deep eutectic solvent-catalyzed glycolysis. Chemosphere.

[12] Achilias D.S., Redhwi H.H., Siddiqui M.N., Nikolaidis A.K., Bikiaris D.N., Karayannidis G.P. (2010). Glycolytic depolymerization of PET waste in a microwave reactor. J. Appl. Polym. Sci..

[13] Liu Y., Zhang C.Z., Feng J., Wang X., Ding Z., He L., Zhang Q., Chen J., Yin Y. (2023). Integrated photochromic-photothermal processes for catalytic plastic upcycling. Angew. Chem..

[14] Liu B., Fu W., Lu X., Zhou Q., Zhang S. (2018). Lewis acid–base synergistic catalysis for polyethylene terephthalate degradation by 1,3-dimethylurea/Zn(OAc)_2_ deep eutectic solvent. ACS Sustain. Chem. Eng..

[15] Zhou L., Lu X., Ju Z., Liu B., Yao H., Xu J., Zhou Q., Hu Y., Zhang S. (2019). Alcoholysis of polyethylene terephthalate to produce dioctyl terephthalate using choline chloride-based deep eutectic solvents as efficient catalysts. Green Chem..

[16] Ju Z., Zhou L., Lu X., Li Y., Yao X., Cheng S., Chen G., Ge C. (2021). Mechanistic insight into the roles of anions and cations in the degradation of poly(ethylene terephthalate) catalyzed by ionic liquids. Phys. Chem. Chem. Phys..

[17] Jang M., Jung E., Yang Y., Noh J., Song H., Kim H., Kang H., Choe S., Choi T.-L., Lee E. (2025). Air and thermally stable cyclic (alkyl)(amino)carbene ruthenium complexes for efficient ring expansion metathesis polymerization. J. Am. Chem. Soc..

[18] Lombardi B.M.P., Faas M.R., West D., Suvinen R.A., Tuononen H.M., Roesler R. (2024). An isolable, chelating bis cyclic (alkyl)(amino)carbene stabilizes a strongly bent, dicoordinate Ni(0) complex. Nat. Commun..

[19] Majumder C., Sharma A., Das B., Yadav R., Kundu S. (2025). Cyclic (alkenyl)(amino)carbene (*^SMe^*CAenAC): Introducing a member to the cyclic (alkyl)(amino)carbenes family featuring a narrow energy gap. J. Am. Chem. Soc..

[20] Yang Y., Jang M., Kang H., Choe S., Lee E., Choi T.-L. (2025). Synthesis of linear and cyclic poly(allenamer)s by powerful cyclic-alkyl-amino-carbene (CAAC) ruthenium catalysts and facile post-modification. Angew. Chem..

[21] Zhou L., Zhang D., Hu J., Wu Y., Geng J., Hu X. (2021). Thermal dehydrogenation and hydrolysis of BH_3_NH_3_ catalyzed by cyclic (alkyl)(amino)carbene iridium complexes under mild conditions. Organometallics.

[22] Hu X., Soleilhavoup M., Melaimi M., Chu J., Bertrand G. (2015). Air-stable (CAAC)CuCl and (CAAC)CuBH_4_ complexes as catalysts for the hydrolytic dehydrogenation of bh_3_nh_3_. Angew. Chem..

[23] Liu P., Hensen E.J. (2013). Highly efficient and robust Au/MgCuCr_2_O_4_ catalyst for gas-phase oxidation of ethanol to acetaldehyde. J. Am. Chem. Soc..

[24] Yang Y., Rioux R.M. (2014). Highly stereoselective anti-markovnikov hydrothiolation of alkynes and electron-deficient alkenes by a supported Cu-NHC complex. Green Chem..

[25] Lima G.R., Monteiro W.F., Ligabue R., Santana R.M.C. (2017). Titanate nanotubes as new nanostrutured catalyst for depolymerization of PET by glycolysis reaction. Mater. Res..

[26] Somayeh M., Bouldo M.G., Mojtaba E. (2023). Controlled glycolysis of poly(ethylene terephthalate) to oligomers under microwave irradiation using antimony(III) oxide. ACS Appl. Polym. Mater..

[27] Sean Najmi D.H., Duncan A., Slanac D., Hutchenson K., Hughes J., Poladi R., Dionisios G. (2025). Vlachos. Closed-loop chemical recycling of polyethylene furan-2,5-dicarboxylate (PEF) under microwave-assisted heating. Green Chem..

[28] Al-Sabagh A.M., Yehia F.Z., Eissa A.M.F., Moustafa M.E., Eshaq G., Rabie A.M., ElMetwally A.E. (2014). Cu- and Zn-acetate-containing ionic liquids as catalysts for the glycolysis of poly(ethylene terephthalate). Polym. Degrad. Stab..

[29] López-Fonseca R., Duque-Ingunza I., de Rivas B., Flores-Giraldo L., Gutiérrez-Ortiz J.I. (2011). Kinetics of catalytic glycolysis of PET wastes with sodium carbonate. Chem. Eng. J..

[30] Sean Najmi B.C.V., Selvam E., Huang D., Dionisios G. (2023). Vlachos. Controlling PET oligomers vs monomers via microwave-induced heating and swelling. Chem. Eng. J..

[31] Toshiaki Yoshioka N.O., Okuwaki A. (1998). Kinetics of hydrolysis of PET powder in nitric acid by a modified shrinking-core model. Ind. Eng. Chem. Res..

[32] Nguyet Thi Ho L., Minh Ngo D., Cho J., Jung H.M. (2018). Enhanced catalytic glycolysis conditions for chemical recycling of glycol-modified poly(ethylene terephthalate). Polym. Degrad. Stab..

[33] Yue Q., Xiao L., Zhang M., Bai X. (2013). The glycolysis of poly(ethylene terephthalate) waste: Lewis acidic ionic liquids as high efficient catalysts. Polymers.

[34] Wang Q., Geng Y., Lu X., Zhang S. (2015). First-row transition metal-containing ionic liquids as highly active catalysts for the glycolysis of poly(ethylene terephthalate) (PET). ACS Sustain. Chem. Eng..

