# Supplementary material for: Efficient Glycolysis of Polyethylene Terephthalate (PET) Catalyzed by Cyclic(alkyl)(amino)carbene Copper Complexes
Source: Molecules. 2025 Nov 23;30(23):4521. doi: 10.3390/molecules30234521 (PMC12692927; doi:10.3390/molecules30234521)
Supplement: Supplementary file 1 [file molecules-30-04521-s001.zip › molecules-3988182-supplementary.pdf]

Efficient Glycolysis of Polyethylene Terephthalate (PET) Catalyzed by Cyclic(alkyl)(amino)carbene Copper Complexes

Supplementary Information

Lei Zhou[1,2,*], Irfan Purnawan[3], Nurul Ffithriyah[3], Mingxin Li[1,2], Hao Huang[1,2], Jiaqin He[1,2] and Yuanyou Wang[1,2,*]

1 School of Chemical Engineering, Yangzhou Polytechnic Institute, Yangzhou 225127, Jiangsu, China; 18362929192@163.com (M.L.); Huangh0825@163.com (H.H.); hejq@ypi.edu.cn (J.H.)

2 Jiangsu Polyester Synthesis and Renewable Technology Engineering Research Center, Yangzhou 225127, Jiangsu, China

3 Chemical Engineering Department, Universitas Muhammadiyah Jakarta, Jakarta 10510, Indonesia; irfan.purnawan@umj.ac.id (I.P.); nurul.fithriyah@umj.ac.id (N.F.)

* Correspondence: zhoulei940528@163.com (L.Z.); 18012336591@163.com (Y.W.)

CAAC-Cu and product analysis

17.7 g (0.10 mol) 2,6-Diisopropylaniline, 10.0 g (0.12 mol) 2-Ethylbutyraldehyde and 12.0 g anhydrous magnesium sulfate (dehydrating agent) were added in to a 250 mL single-bottle flask, then the mixture was dissolved in 100 mL dichloromethane. The reaction was stirred at room temperature for 24 h. After the reaction, undissolved compounds were filtered. Then, the solvent was removed by rotary evaporation, and the target product was obtained by vacuum drying at 50 ℃ overnight. Under the protection of nitrogen, 20 g (0.11 mol) product was dissolved in 50 mL anhydrous ethyl ether and cooled to -78 ℃. Under the magnetic agitation, 50 mL n-Butyllithium solution (concentration: 2.5 M in hexyl hydride) was slowly added with a syringe. The solution was kept at -78 ℃ for 0.5 h, then raised to room temperature and stirred for 1 h. The reaction solution was cooled to -78 ℃, and 13.9 g (0.15 mol) 3-Chloro-2-methylpropene was slowly added with a syringe under magnetic agitation. The mixture was raised to room temperature and stirred for 24 h to obtain a white suspension. The volatiles were removed by vacuum at room temperature to produce a solid mixture. The solid mixture was added with 50 mL anhydrous ether under nitrogen protection, cooled to -78 ℃, and 154 mL HCl ether solution (1 M) was added under agitation for 2h. Then, the solution was transferred to a sealed reactor and stirred for 48h at 85 ℃. After filtration, using anhydrous ether to wash the mixture several times to produce white solid. Then, the white solid was added into an aqueous solution containing 15 g (0.14 mol) $NaBF_4$, and white precipitation CAAC was obtained through filtration. After dried under vacuum, CAAC was obtained as a white solid (24.8 g, 63% yield).

In a Schlenk flask, 2.0 g (5 mmol) CAAC, 1.05 g (5.25 mmol) Potassium bis(trimethylsilyl)amide (KHMDS) and 0.6 g (6 mmol) copper(I) chloride were added. Under the protection of nitrogen at -78 ℃, 50 mL anhydrous THF was injected slowly by a syringe. The solution was stirred at -78 ℃ for 30 mins. Then, it was slowly heated to room temperature and stirred overnight. The volatiles were evaporated under vacuum and the residue was washed with hexane (40 mL). After removing the volatile, the residue

was extracted with benzene (60 mL). The solution was evaporated and dried under vacuum, affording a white solid. CAAC-Cu (1.25 g, 57 % yield).

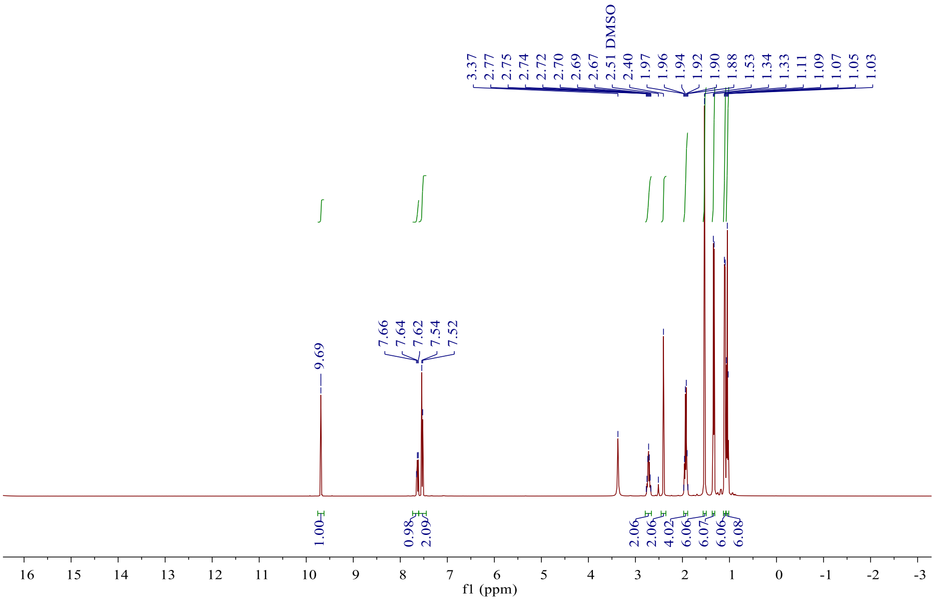

Figure S1. ^{1}H NMR spectrum of CAAC

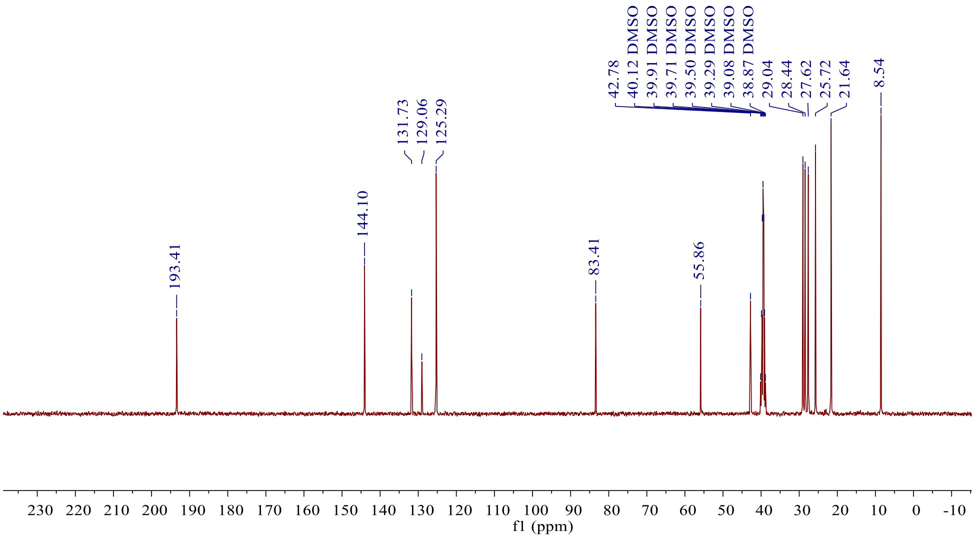

Figure S2. ^{13}C NMR spectrum of CAAC

[CAAC] ^{1}H NMR (DMSO, 400 MHz, δ): 9.69 (s, 1H, CH=N), 7.64 (t, 1H, CH^{Ar}), 7.54 (d, 2H, CH^{Ar}), 2.72 (m, 2H, $CH^{i\text{-}pr}$), 2.40 (s, 2H, CH_2), 1.94 (m, 4H, CH_2^{Et}), 1.53 (s, 6H, CH_3), 1.34 (d, 6H, CH_3^{Et}), 1.11 (d, 6H, $CH_3^{i\text{-}pr}$), 1.05 (t, 6H, $CH_3^{i\text{-}pr}$). ^{13}C NMR (DMSO, 100 MHz, δ): 193.41 (C, CH=N), 144.10, 131.73, 129.06, 125.29 (C, Ar), 83.41, 55.86 (C, quaternary carbon), 42.78 (CH_2), 29.04 (CH_2, Et), 28.44 (CH, Dipp), 27.62 (CH_3), 25.72, 21.64 (CH_3, Dipp), 8.54 (CH_3, Et).

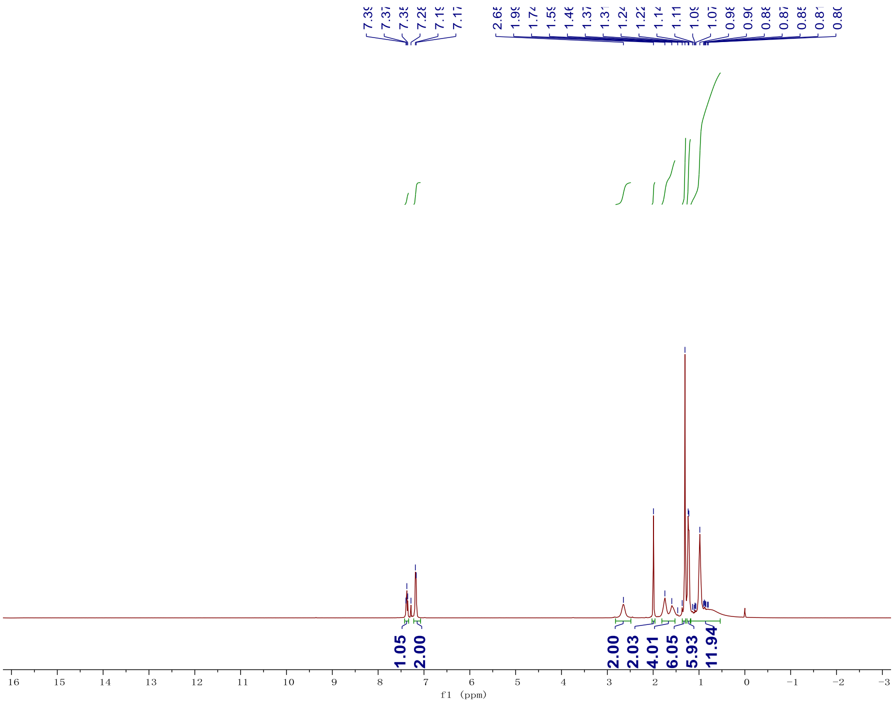

Figure S3. ^{1}H NMR spectrum of CAAC-Cu

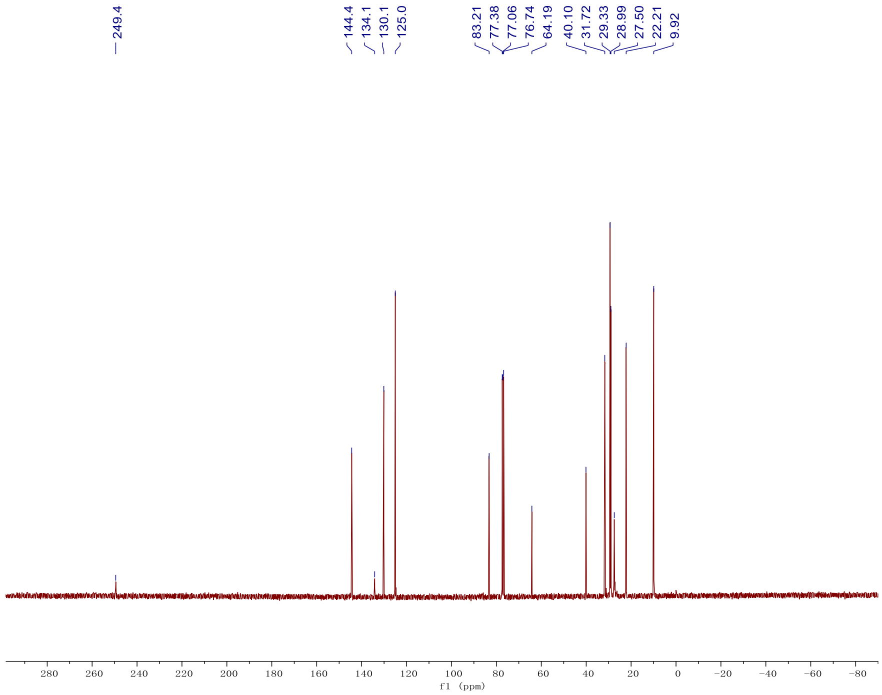

Figure S4. ^{13}C NMR spectrum of CAAC-Cu

[CAAC-Cu] ^{1}H NMR (400 MHz, $CDCl_3$) δ 7.37 (t, *J* = 7.2 Hz, 1H), 7.19 (d, *J* = 7.2 Hz, 2H), 2.65 (s, 2H), 1.99 (s, 2H), 1.37-1.74 (m, *J* = 7.2 Hz, 4H), 1.31-1.22 (m, *J* = 7.2 Hz, 6H), 1.09-1.14 (m, *J* = 7.2 Hz, 6H), 0.80-1.07 (m, *J* = 6.9 Hz, 12H). ^{13}C NMR (101 MHz, $CDCl_3$) δ 249.48 (C, carbene), 144.41 (C, Ar), 134.17 (C, Ar), 130.11 (C, Ar), 125.01 (C, Ar), 83.21 (Cq), 64.19 (Cq), 40.10 (CH_2), 31.72 (CH_2), 29.33 (CH_3), 28.99 (CH_3), 27.50 (CH), 22.21 (CH_3), 9.92 (CH_3).

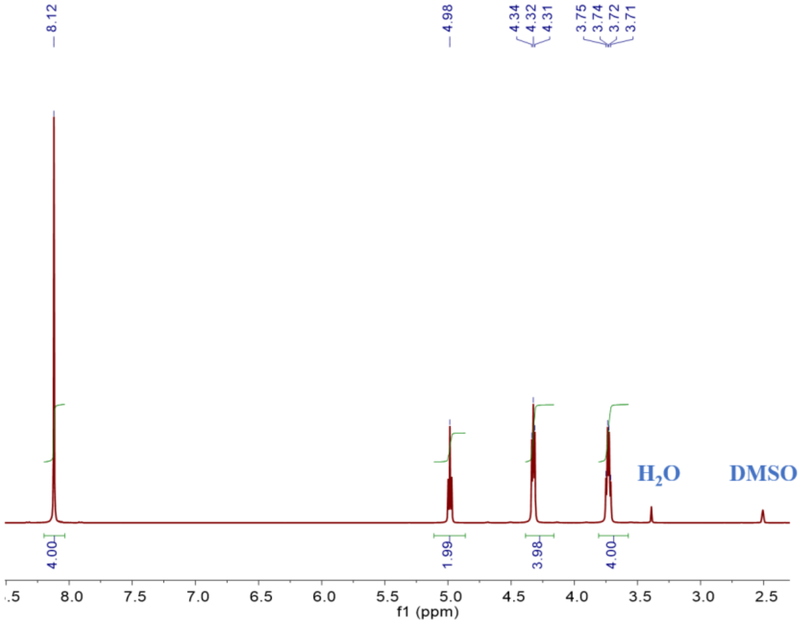

Figure S5. ^{1}H NMR spectrum of the main product

The result of ^{1}H NMR was shown in Figure S5, ^{1}H NMR (DMSO, 400 MHz), δ 8.12 (s, 4H, Ar), 4.98 (s, 2H, OH), 4.39 – 4.22 (m, 4H, CH_2), 3.73 (q, 4H, CH_2).

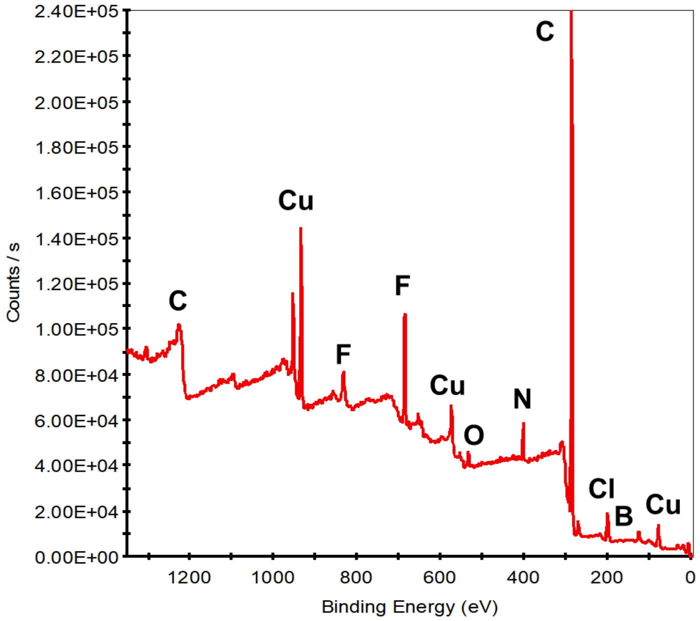

Figure S6. XPS spectrum of CAAC-Cu was calibrated using the C 1s peak of carbon at 284.5 eV.

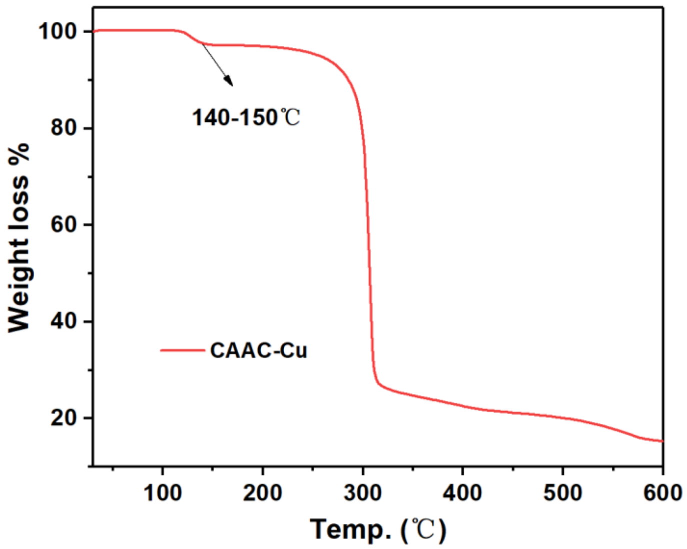

Figure S7. TGA curves of CAAC-Cu

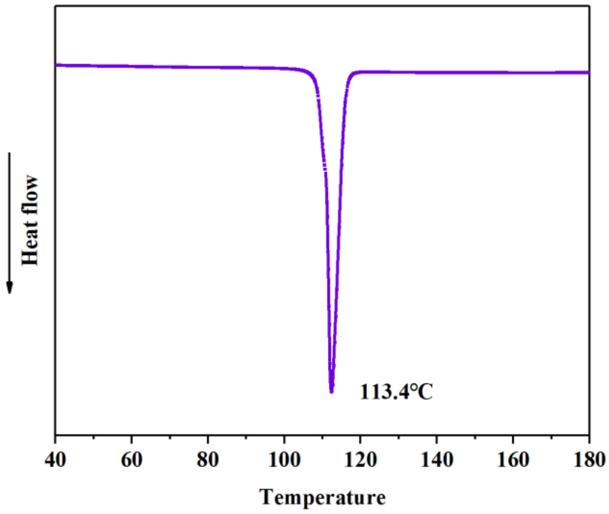

Figure S8. DSC curves of main product

Figure S8 presents the DSC curve of the primary product, which exhibits a distinct sharp endothermic peak. The temperature at which melting initiates is recorded as 113.4 °C, a value that aligns well with the known melting point properties of BHET.

Table S1. Elemental analysis results of the main product

	C %	H %	O %
Theoretical value	56.67	5.56	37.92
Main product	56.74	5.62	37.88

Table S2. Recycling experiment data

Recycle numbers	Degradation means	Standard deviation	Yield means	Standard deviation

1	97.7	1.58219	85.8	1.75594
2	96.9	1.65227	85.3	2.20303
3	95.1	2.00333	83.9	2.10079
4	94.2	1.85203	83.2	1.82455
5	94.3	1.75214	82.5	1.40475

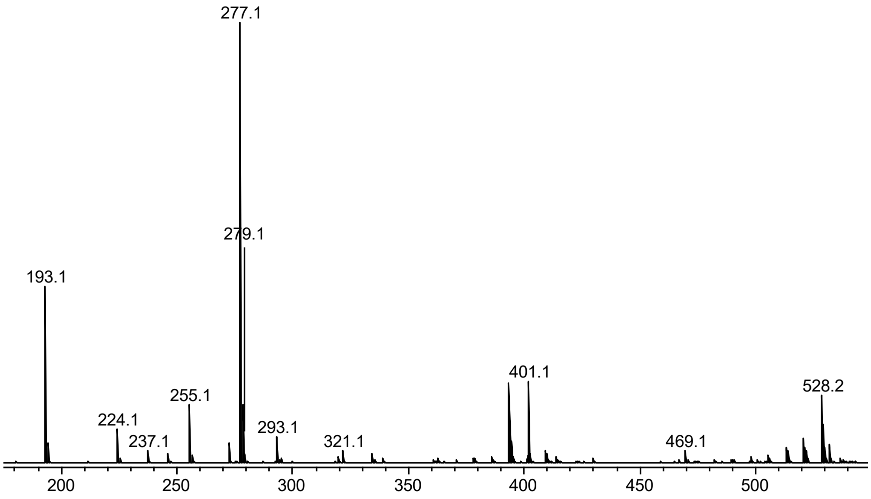

Figure S9. Mass spectrum of main product

The result of mass spectrometry was shown in Figure S9, MS (m/z): $[M]^+$ calcd for $[BHET+Na]^+$, 277.07; found, 277.10. Further confirmed that the product is BHET.
